# Identification of transcriptional networks controlling leaf sheath growth in *Sorghum bicolor*

**DOI:** 10.1186/s13104-023-06653-z

**Published:** 2024-01-02

**Authors:** Samuel De Riseis, Frank G. Harmon

**Affiliations:** 1 Plant Gene Expression Center, USDA-ARS, Albany, 94710 USA; 2 Department of Plant & Microbial Biology, University of California, Berkeley, CA 94720 USA

**Keywords:** *Sorghum bicolor*, FASTQ file, Illumina sequencing, RNAseq, Leaf sheath, Seedling stem

## Abstract

**Objectives:**

The objective of this data set was to identify transcriptional networks that control elongation of seedling leaf sheaths in the C4 grass *Sorghum bicolor*. One motivation was that leaf sheaths are a primary constituent of stems in grass seedlings; therefore, genes that control growth of this organ are important contributors to successful transition from the seedling stage to the mature plant stage and, ultimately, crop success. Since diurnal rhythms contribute to regulation of signaling networks responsible for growth, a time course representing the late afternoon and early evening was anticipated to pinpoint important control genes for stem growth. Ultimately, the expected outcome was discovery of transcript networks that integrate internal and external signals to fine tune leaf sheath growth and, consequently, plant height.

**Data description:**

The data set is RNAseq profiling of upper leaf sheaths collected from wild type *Sorghum bicolor* (BTx623 line) plants at four-hour intervals from 12.5 h after dawn to 20 h after dawn. Global transcript levels in leaves were determined by deep sequencing of mRNA from four individual seedlings at each time point. This data set contains sequences representing the spectrum of mRNAs from individual genes. This data set enables detection of significant changes in gene-level expression caused by the progression of the day from late afternoon to the middle of the night. This data set is useful to identify gene expression networks regulating growth in the leaf sheath, an organ that is a major contributor to the sorghum seedling stem and defines seedling height.

## Objective

Sorghum is in the family *Poaceae* that includes the other important agricultural crops maize, sugarcane, rice, and wheat. The grass leaf is composed of several distinct structures, including the upper portion known as the blade and the lower portion known as the sheath. The leaf sheath wraps around emerging immature leaves and internodes from which leaves originate. The stem in young seedlings is primarily composed of successive leaf sheaths and, therefore, these organs are critical for structural support and to establish plant height. The amount of elongation occurring in the sheath depends internal and external signals that are incompletely understood. The objective of this data set was to identify transcriptional networks that control elongation of the leaf sheath in *Sorghum bicolor* as a representative of the Poaceae family. Since diurnal rhythms are well known to contribute to regulation of growth-related signaling networks, a time course representing the late afternoon and early evening was expected to highlight likely control genes for stem growth. Ultimately, the expected outcome was discovery of transcript networks that integrate internal and external signals to fine tune leaf sheath growth and, consequently, plant height. These data are useful for engineering plant stature and to alter the sensitivity of growth in cereal crops to the consequences of environmental change.

## Data description

The data set is RNAseq of the top half of the leaf 5 sheath from individual wild type Sorghum bicolor (BTx623 inbred, carrying the *male sterile 8* (*ms8*) mutation [1] seedlings. Plants were grown in 4-inch peat pots filled with SuperSoil (The Scotts Company) soil under greenhouse conditions of 16-hour days and 8-hour nights. Natural sunlight was supplemented with LumiGrow Pro325 LEDs. Daytime temperature was set to 26°C and nighttime temperature was set to 20°C. After 14 days, the plants were transferred to Percival growth chambers set to 16 hours of white LED light, at 360 µmol photons m^-1^s^-1^, and 8 hours of darkness, with daytime temperature set to 26°C and nighttime temperature set to 22°C. Samples were dissected from 21-day-old plants by removing leaves 1–4, cutting the leaf 5 sheath in the middle, and the sheath tissue unwrapped from the underlying leaves. Samples were immediately frozen in liquid nitrogen. Plants were sampled at 12.5, 16, and 20 hours after dawn in three biological replicates for a total of 4 samples for each time point, totaling 12 samples. Total RNA for each individual was isolated with the Qiagen Plant RNeasy Kit (www.qiagen.com) according to manufacturer’s recommendations. Residual genomic DNA was removed by on-column digestion with the Qiagen RNase-Free DNA Set (www.qiagen.com) according to manufacturer’s recommendations. Sequencing library preparation and Illumina NovaSeq 6000 (www.illumina.com) next generation sequencing was done by Novogene Corporation Inc. (www.novogene.com/us-en/). Nondirectional libraries were prepared with the “NEBNext Ultra II RNA Library Prep Kit for Illumina” from New England Biolabs (www.neb.com) with messenger RNA purified from total RNA using poly-T oligo-attached magnetic beads. Library concentration quantification used Qubit and real-time PCR. Fragment size distribution was determined by Bioanalyzer (Agilent Technologies, www.agilent.com). Pooled libraries were pair-end 150 base pair sequenced on one lane of Illumina NovoSeq 6000. Raw reads were filtered for adapter sequences and low-quality reads based on 1) reads containing N > 10% (N represents the base cannot be determined) or 2) reads containing low quality (Q-score < = 5) bases which is over 50% of the total bases. 5’ adapter sequence: 5’-AGATCGGAAGAGCGTCGTGTAGGGAAAGAGTGTAGATCTCGGTGGTCGCCGTATCATT-3’ and 3’ adapter sequence: 5’-GATCGGAAGAGCACACGTCTGAACTCCAGTCACGGATGACTATCTCGTATGCCGTCTTCTGCTTG-3’. These pass-filter reads were demultiplexed according to the 12 biological samples into forward read and reverse read FASTQ files. The total number of pass filter reads for each data set were as follows: 63,620,858 in data set 1, 44,181,504 in data set 2, 39,214,778 in data set 3, 61,216,552 in data set 4, 45,273,096 in data set 5, 54,439,560 in data set 6, 55,008,376 in data set 7, 62,795,986 in data set 8, 41,410,692 in data set 9, 47,362,084 in data set 10, 45,503,020 in data set 11, and 54,403,120 in data set 12. The FASTQ files were deposited at the National Center for Biotechnology (NCBI) as BioProject ID PRJNA1008758 (https://identifiers.org/ncbi/bioproject:PRJNA1008758) [2].

**Table 1:** Overview of data files/data sets

Label	Name of data file/data set	File types (file extension)	Data repository and identifier (DOI or accession number)
Data set 1	RNAseq of wild type leaf sheath collected at ZT 12.5, replicate 1	.FASTQ	NCBI Sequence Read Archive (https://identifiers.org/ncbi/insdc.sra:SRR25749286) [3]
Data set 2	RNAseq of wild type leaf sheath collected at ZT 12.5, replicate 2	.FASTQ	NCBI Sequence Read Archive (https://identifiers.org/ncbi/insdc.sra:SRR25749285) [4]
Data set 3	RNAseq of wild type leaf sheath collected at ZT 12.5, replicate 3	.FASTQ	NCBI Sequence Read Archive (https://identifiers.org/ncbi/insdc.sra:SRR25749282) [5]
Data set 4	RNAseq of wild type leaf sheath collected at ZT 12.5, replicate 4	.FASTQ	NCBI Sequence Read Archive (https://identifiers.org/ncbi/insdc.sra:SRR25749281) [6]
Data set 5	RNAseq of wild type leaf sheath collected at ZT 16, replicate 1	.FASTQ	NCBI Sequence Read Archive (https://identifiers.org/ncbi/insdc.sra:SRR25749280) [7]
Data set 6	RNAseq of wild type leaf sheath collected at ZT 16, replicate 2	.FASTQ	NCBI Sequence Read Archive (https://identifiers.org/ncbi/insdc.sra:SRR25749279) [8]
Data set 7	RNAseq of wild type leaf sheath collected at ZT 16, replicate 3	.FASTQ	NCBI Sequence Read Archive (https://identifiers.org/ncbi/insdc.sra:SRR25749278) [9]
Data set 8	RNAseq of wild type leaf sheath collected at ZT 16, replicate 4	.FASTQ	NCBI Sequence Read Archive (https://identifiers.org/ncbi/insdc.sra:SRR25749277) [10]
Data set 9	RNAseq of wild type leaf sheath collected at ZT 20, replicate 1	.FASTQ	NCBI Sequence Read Archive (https://identifiers.org/ncbi/insdc.sra:SRR25749276 [11]
Data set 10	RNAseq of wild type leaf sheath collected at ZT 20, replicate 2	.FASTQ	NCBI Sequence Read Archive (https://identifiers.org/ncbi/insdc.sra:SRR25749275) [12]
Data set 11	RNAseq of wild type leaf sheath collected at ZT 20, replicate 3	.FASTQ	NCBI Sequence Read Archive (https://identifiers.org/ncbi/insdc.sra:SRR25749278) [9]
Data set 12	RNAseq of wild type leaf sheath collected at ZT 20, replicate 4	.FASTQ	NCBI Sequence Read Archive (https://identifiers.org/ncbi/insdc.sra:SRR25749283) [13]

### Limitations

- The leaf sheath is a specific leaf structure. This should be considered if these data are interpreted for other parts of the leaf, as well as for different plant tissues.
- The sample collection strategy taken here, which focused on gene expression in the afternoon to evening hours, may result in under representation of transcripts primarily expressed at other parts of the day.
- The RNAseq libraries were non-stranded. As a consequence, these libraries represent overall gene expression, but do not discriminate between sense and antisense transcripts.

## Data Availability

All the FASTQ files described in this Data Note can be freely and openly accessed at the NCBI Sequence Read Archive (SRA) under accession numbers SRR25749286, SRR25749285, SRR25749282, SRR25749281, SRR25749280, SRR25749279, SRR25749278, SRR25749277, SRR25749276, SRR25749275, SRR25749278, and SRR25749283 with the resolving links in Table 1 [3–13]. All data and details on the samples are gathered together under NCBI BioProject ID PRJNA1008758 (https://identifiers.org/ncbi/bioproject:PRJNA1008758) [2].

## Abbreviations

mRNA: messenger RNA
ms8: male sterile 8
NCBI: National Center for Biotechnology Information
Q-score: Illumina quality score
SRA: Sequence Read Archive
ZT: Zeitgeber Time
